# A Novel Cell Delivery System Exploiting Synergy between Fresh Titanium and Fibronectin

**DOI:** 10.3390/cells11142158

**Published:** 2022-07-10

**Authors:** Makoto Hirota, Norio Hori, Yoshihiko Sugita, Takayuki Ikeda, Wonhee Park, Juri Saruta, Takahiro Ogawa

**Affiliations:** 1 Division of Regenerative and Reconstructive Sciences and Weintraub Center for Reconstructive Biotechnology, UCLA School of Dentistry, Los Angeles, CA 90095-1668, USA; mhirota@yokohama-cu.ac.jp (M.H.); horinorio@gmail.com (N.H.); yosshii@dpc.agu.ac.jp (Y.S.); ikeda.takayuki@nihon-u.ac.jp (T.I.); drwon69@gmail.com (W.P.); saruta@kdu.ac.jp (J.S.); 2 Department of Oral and Maxillofacial Surgery/Orthodontics, Yokohama City University Medical Center, 4-57 Urafune-cho, MInami-ku, Yokohama 232-0024, Kanagawa, Japan; 3 Department of Oral Pathology, School of Dentistry, Aichi Gakuin University, 1-1-100 Kusumoto-cho, Chikusa-ku, Nagoya 464-8650, Aichi, Japan; 4 Department of Complete Denture Prosthodontics, Nihon University School of Dentistry, 1-8-13 Kanda Surugadai, Chiyoda-ku, Tokyo 101-8310, Japan; 5 Department of Dentistry, College of Medicine, Hanyang University, 222 Wangsimni-ro, Seongdong-gu, Seoul 04763, Korea; 6 Department of Education Planning, School of Dentistry, Kanagawa Dental University, 82 Inaoka, Yokosuka 238-8580, Kanagawa, Japan

**Keywords:** biological aging of titanium, bone engineering, implants, osseointegration, osteoblasts

## Abstract

Delivering and retaining cells in areas of interest is an ongoing challenge in tissue engineering. Here we introduce a novel approach to fabricate osteoblast-loaded titanium suitable for cell delivery for bone integration, regeneration, and engineering. We hypothesized that titanium age influences the efficiency of protein adsorption and cell loading onto titanium surfaces. Fresh (newly machined) and 1-month-old (aged) commercial grade 4 titanium disks were prepared. Fresh titanium surfaces were hydrophilic, whereas aged surfaces were hydrophobic. Twice the amount of type 1 collagen and fibronectin adsorbed to fresh titanium surfaces than aged titanium surfaces after a short incubation period of three hours, and 2.5-times more fibronectin than collagen adsorbed regardless of titanium age. Rat bone marrow-derived osteoblasts were incubated on protein-adsorbed titanium surfaces for three hours, and osteoblast loading was most efficient on fresh titanium adsorbed with fibronectin. The number of osteoblasts loaded using this synergy between fresh titanium and fibronectin was nine times greater than that on aged titanium with no protein adsorption. The loaded cells were confirmed to be firmly attached and functional. The number of loaded cells was strongly correlated with the amount of protein adsorbed regardless of the protein type, with fibronectin simply more efficiently adsorbed on titanium surfaces than collagen. The role of surface hydrophilicity of fresh titanium surfaces in increasing protein adsorption or cell loading was unclear. The hydrophilicity of protein-adsorbed titanium increased with the amount of protein but was not the primary determinant of cell loading. In conclusion, the osteoblast loading efficiency was dependent on the age of the titanium and the amount of protein adsorption. In addition, the efficiency of protein adsorption was specific to the protein, with fibronectin being much more efficient than collagen. This is a novel strategy to effectively deliver osteoblasts ex vivo and in vivo using titanium as a vehicle.

## 1. Introduction

Cells, growth factors, and scaffolds are a necessary triad for tissue engineering. Despite advances in scaffold development and the identification of new growth factors and their genetic manipulation [1,2,3,4,5,6,7,8,9,10,11,12,13,14], delivering sufficient numbers of cells and maintaining them in areas of interest, regardless of whether for ex vivo tissue engineering or in situ regeneration, has been challenging [1,12,15,16,17,18,19,20]. The effective delivery of stem cells or osteogenic cells derived from bone marrow or other source organs has been investigated in the context of tissue engineering and regeneration [12,21,22,23,24,25,26,27,28,29,30,31,32,33,34,35,36,37,38,39,40,41,42,43,44,45]. Various methods to improve cellular delivery have also been examined, including cell sheet technology, injectable vehicles, carrier molecules, and 3-dimensional (3D) cell printing [2,27,29,46,47,48,49,50,51,52]. Despite some progress, cell-based repair and regeneration of tissues, particularly fractured and diseased bone remains far from being a standard clinical modality, with the approach hampered by poor clinical viability, high costs, and suboptimal biological efficacy [37,38,47,53,54,55,56,57,58,59].

Among the tissue engineering triad, scaffolds or biomaterials play a critical role not just as a framework of the tissue but also as a delivery vehicle for cells. Various biomaterials such as collagen [60,61], gelatin [62], hydrogel/alginate [63,64], chitosan [65,66,67], and hyaluronic acid [68], have been utilized to effectively regenerate various soft tissues including nerve [69,70], skin [71], cartilage [66,72], and vascular tissue [73]. With regard to bone engineering, metal-based solid materials have been used mostly to meet the mechanical requirements of the tissue. Titanium is a biocompatible and chemically stable metal that has long been used as an orthopedic and dental implant material and, more recently, as a scaffold for bone regeneration and engineering [27,28,39,40,41,42,43,44,45,74,75,76,77,78,79,80,81,82,83,84,85,86,87,88,89,90,91,92,93]. Titanium is innately osteoconductive, but accelerated osseointegration (bone-implant integration) and ensuring osseointegration under unfavorable local and systemic conditions remain challenging. Overall, it has been very difficult to recruit osteogenic cells to titanium scaffolds made of thin fibers and porous or complex structures for titanium-driven bone regeneration and engineering [18,27,28,41,42,43,44]. Further, to meet the demands of load-bearing, titanium implants must form bone in direct contact with the implant. Therefore, success depends not only on the effective delivery of cells around titanium but also on locating and retaining cells close to the implant surface or at the bone-implant interface.

The concept of biological aging of titanium is based on the discovery that the hydrophilicity of titanium decreases over time, such that titanium surfaces used under ordinary conditions (both clinically and experimentally) are hydrophobic, whereas fresh titanium surfaces (i.e., immediately after machining or etching) are hydrophilic [94,95,96,97,98,99,100,101,102,103,104,105,106,107,108,109]. These time-dependent changes in hydrophilicity are also accompanied by time-dependent changes in biological properties [78,99,104,110,111,112,113]. Therefore, to develop a novel cell delivery system using titanium as a carrier, we examined the combined effect of titanium age and protein adsorption to titanium as a cell attachment mediator on the efficacy of osteoblast loading onto the titanium surfaces. Two different extracellular matrix proteins, collagen 1 and fibronectin, were used to promote osteoblast loading. The role of hydrophilicity of titanium on the efficacy of protein adsorption and cell loading was thoroughly studied.

## 2. Materials and Methods

### 2.1. Titanium Samples and Surface Characterization

Titanium experimental samples in disk form (20 mm diameter, 1.0 mm thickness) were machine-prepared from grade 4 commercially pure titanium. All the disks were cleaned with 70% ethanol and ddH_2_O, and sterilized with gamma rays. Half of the disks were used for testing immediately (fresh surfaces) and the other half were placed in a sealed container and stored in a dark room (temperature, 23 °C; humidity, 60%) for one month (aged surfaces). The surface morphology was examined by scanning electron microscopy (SEM; Nova 230 Nano SEM, FEI, Hillsboro, OR, USA) and an optical profile microscope (MeX, Alicona Imaging GmbH, Raaba, Graz, Austria) for three-dimensional imaging and quantitative roughness analysis. The average roughness (Sa) and peak-to-valley roughness (Sz) were calculated. The hydrophilicity/hydrophobicity of the fresh and aged titanium surfaces with and without protein adsorption was measured using an automated contact angle measuring device (DCA-VZ, Kyowa, Interface Science, Saitama, Japan) as the contact angle of 1 µL of ddH_2_O.

### 2.2. Protein Adsorption

Bovine serum albumin, fraction V (Pierce Biotechnology, Inc., Rockford, IL, USA) was used as a model protein. A previously established bicinchoninic acid (BCA)-based colorimetric detection (Micro BCA Protein Assay Kit, Thermo Scientific, Rockford, IL, USA) was used [94,97,98]. One hundred µL of protein solution (1 mg/mL protein/saline) was spread over a disk. After 24 h of incubation in sterile humidified conditions at 37 °C, the solution containing nonadherent proteins was removed and mixed with microbicinchoninic acid (Pierce Biotechnology) at 37 °C for 60 min. The amount of the removed protein was quantified using a microplate reader (Synergy HT, BioTek Instruments, Winooski, VT, USA) at 562 nm. The rate of protein adsorption was calculated as the percentage of proteins adsorbed to titanium surfaces relative to the total amount inoculated.

### 2.3. Osteoblast Cell Culture

As described elsewhere [80,89,108,114,115,116], bone marrow-derived osteoblasts were isolated from the femurs of 8-week-old male Sprague–Dawley rats and placed into alpha-modified Eagle’s medium supplemented with 15% fetal bovine serum, 50 µg/mL ascorbic acid, 10 mM Na-β-glycerophosphate, 10^−8^ M dexamethasone, and antibiotic–antimycotic solution containing 10,000 units/mL penicillin G sodium, 10,000 mg/mL streptomycin sulfate, and 25 mg/mL amphotericin B. The cells were incubated in a humidified atmosphere of 95% air and 5% CO_2_ at 37 °C. At 80% confluency, the cells were detached using 0.25% trypsin–1 mM EDTA-4Na and seeded onto titanium disks placed in 12-well culture dishes at a density of 3 × 10^4^ cells/cm^2^. The density was established in our previous studies for bone marrow-derived osteoblasts extracted from young rats and has been used with a minor modification [45,80,81,82,83,89,117,118,119,120].

### 2.4. Osteoblast Attachment and Settling Behavior

The level of osteoblast cell attachment to titanium surfaces during a 3 h incubation was evaluated by measuring the quantity of cells attached to titanium surfaces using a hematocytometer. Three hours after seeding, the cells were gently rinsed twice with PBS and treated with 0.1% collagenase in 300 µL of 0.25% trypsin,1 mM EDTA-4Na for 15 min at 37 °C. The number of detached cells was counted. The initial reaction of cells on titanium surfaces was evaluated by examining spreading behavior and the expression of the cytoskeletal and adhesion proteins under a confocal laser scanning microscopy (CLSM) (TCS SP5, Leica, Wetzlar, Germany). The cells were fixed with 10% formalin and stained with rhodamine-phalloidin dye (actin filament, red color; R415, Molecular Probes, Eugene, OR, USA) and vinculin (green color; ab11194, Abcam, Cambridge, UK).

### 2.5. Alkaline Phosphatase (ALP) Activity

The ALP activity of the attached osteoblasts was examined using staining-based and chemical detection-based assays. For the staining-based assay [45,80,95,121,122,123,124,125,126], cultured osteoblasts were washed twice with Hank’s solution and then incubated with 120 mM Tris buffer (pH 8.4) containing 0.9 mM naphthol AS-MX phosphate and 1.8 mM fast red TR for 30 min at 37 °C. For colorimetric detection, the cultured cells were rinsed with ddH_2_O, and 250 µL of p-nitrophenyl phosphate was added, followed by incubation at 37 °C for 15 min. The ALP activity was evaluated by measuring the released nitrophenol in the enzymatic reaction and determined at 405 nm using a plate reader.

### 2.6. Statistical Analyses

The data on surface roughness parameters were collected from five sites on two different disks (*n* = 5). Three disks were used for all the contact angle measurement, protein adsorption and cell culture studies (*n* = 3). A two-way ANOVA was performed to examine the effect of titanium age and protein adsorption. When appropriate, Bonferroni’s test was used as a post hoc test. *p*-values less than 0.05 were considered statistically significant. Regression analysis was extensively applied to determine the correlation among surface factors, protein adsorption, and the number of cells loaded on titanium surfaces.

## 3. Results

### 3.1. Surface Topography and Hydrophilic/Hydrophobic State of Titanium

**Figure 1:**
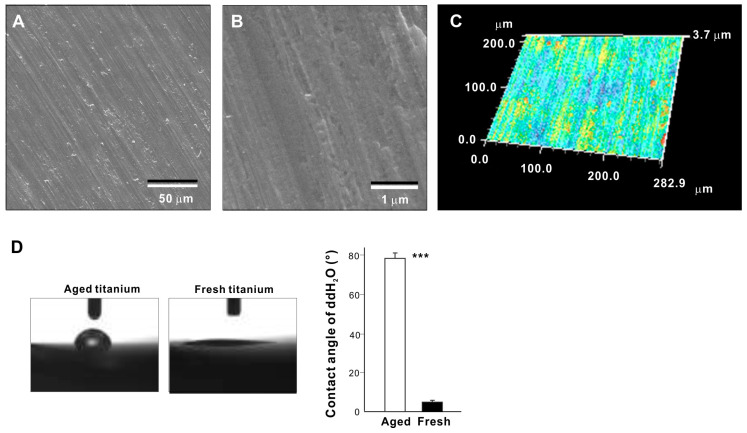
Surface characterization of titanium disks used in this study. Low (**A**) and high (**B**) magnification SEM images of the titanium disks. (**C**) 3-dimensional imaging of the titanium disks obtained with an optical profilometer. (**D**) Side-view photographs of a 1 µL drop of ddH_2_O placed on a titanium disk and a histogram showing contact angle measurements. Fresh (newly prepared) titanium and aged (stored for 1 month) titanium disks were compared. *** *p* < 0.001, statistically significant difference between fresh and aged titanium surfaces.

The machined-surfaced titanium disks used in this study showed concentric marks and other irregularities from machine turning in SEM images (Figure 1A,B) and 3D imaging with an optical profilometer (Figure 1C). The average roughness (Sa) and peak-to-valley roughness (Sz) were 0.23 ± 0.02 µm and 3.42 ± 0.25 µm, respectively. The aged titanium surfaces (one month old) were hydrophobic with a ddH_2_O contact angle of 78.3° ± 1.9°, whereas freshly turned (fresh) titanium surfaces were hydrophilic with a ddH_2_O contact angle of 5.3° ± 0.8° (Figure 1D).

### 3.2. Protein Adsorption Efficiency

**Figure 2:**
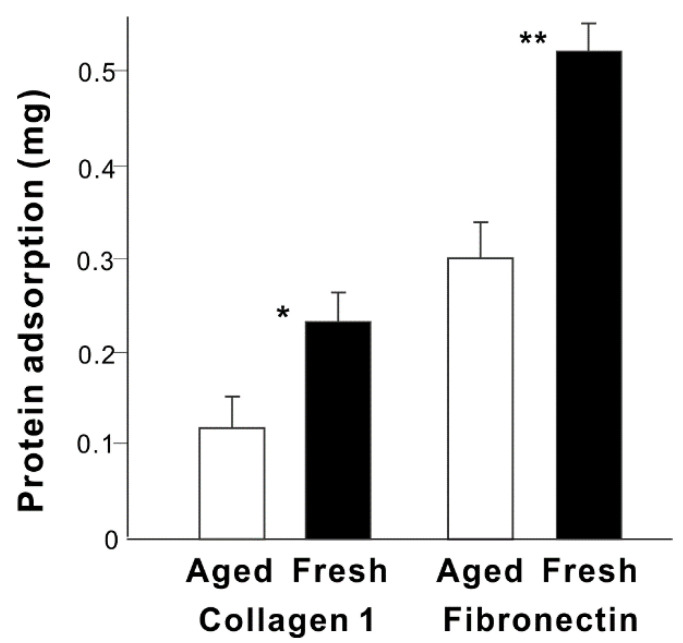
The ability of fresh and aged titanium surfaces to adsorb different proteins. The amount of protein adsorbed to titanium disks during a 3-h incubation is shown. * *p* < 0.05, ** *p* < 0.01, statistically significant difference between fresh and aged titanium surfaces.

We next examined possible differences in protein adsorption onto fresh and aged titanium surfaces with differing hydrophilicity/hydrophobicity (Figure 2). Twice the amount of type 1 collagen and 1.8-times more fibronectin adsorbed to fresh titanium surfaces after a short, 3-h incubation period than to aged titanium surfaces. Regardless of age, the titanium adsorbed 2–2.5 times more fibronectin than collagen.

### 3.3. Change in Hydrophilic/Hydrophobic Status with Protein Adsorption

**Figure 3:**
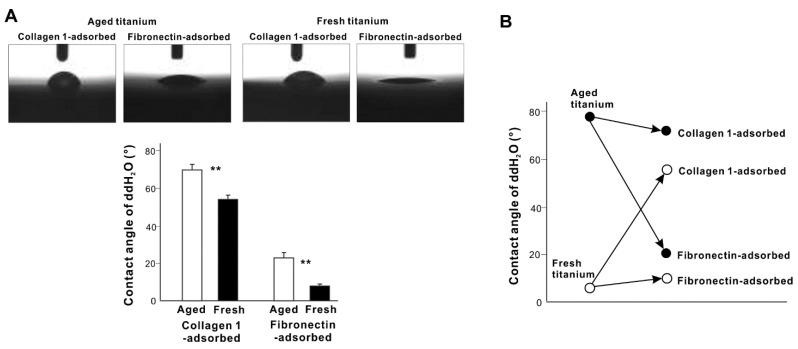
Hydrophobic/hydrophilic state of titanium disks with protein pre-adsorption. (**A**) Side-view photographs of a 1 µL drop of ddH_2_O placed on a protein-pre-adsorbed titanium disk and a histogram showing contact angle measurements. ** *p* < 0.01, statistically significant difference between fresh and aged titanium surfaces. (**B**) Summary of hydrophobic/hydrophilic changes before and after protein adsorption based on Figure 1D and (**A**). Surfaces with a contact angle of ≥40° are defined as hydrophobic, and surfaces with a contact angle < 40° are defined as hydrophilic.

We next examined whether the hydrophilic/hydrophilic status changed after 3 h of protein adsorption (Figure 3A). After adsorbing collagen, the ddH_2_O contact angle decreased on the aged titanium surfaces and increased on the fresh titanium surfaces, although the contact angle was smaller on fresh surfaces than aged surfaces and was considered hydrophobic (contact angle > 40°). Fibronectin-adsorbed titanium surfaces had considerably smaller contact angles than collagen 1-adsorbed titanium surfaces and were hydrophilic, regardless of age (contact angle < 40°). Interestingly, both proteins promoted hydrophilicity when the original contact angle was high, and demoted hydrophilicity when the original contact angle was low (Figure 3B).

### 3.4. Ability of Various Titanium Surfaces to Load Osteoblasts

**Figure 4:**
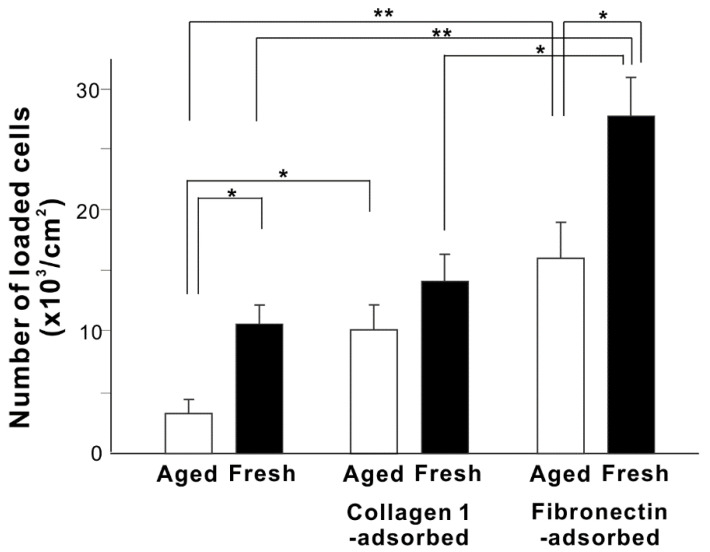
The ability of various titanium surfaces to load osteoblasts. The number of osteoblasts attached to titanium surfaces during a 3-h incubation was measured. Fresh and aged titanium surfaces with or without pre-adsorption of collagen 1 or fibronectin were compared. * *p* < 0.05, ** *p* < 0.01, statistically significant difference between two different surfaces.

We next evaluated osteoblast loading onto aged and fresh titanium surfaces after short-term (3 h) incubation with or without collagen or fibronectin pre-adsorption for 3 h (Figure 4). After 3 h of incubation with cells, fresh titanium surfaces recruited over three times more osteoblasts than aged titanium surfaces. Collagen increased cell loading onto aged titanium surfaces but not fresh titanium surfaces, whereas fibronectin significantly increased cell loading onto both aged and fresh titanium surfaces. Fibronectin-adsorbed fresh titanium surfaces loaded twice the number of osteoblasts compared with collagen-adsorbed fresh titanium surfaces. Fibronectin-adsorbed fresh titanium surfaces loaded the most osteoblasts, approximately nine times more than baseline aged titanium.

### 3.5. Behavior and Function of Loaded Osteoblasts

**Figure 5:**
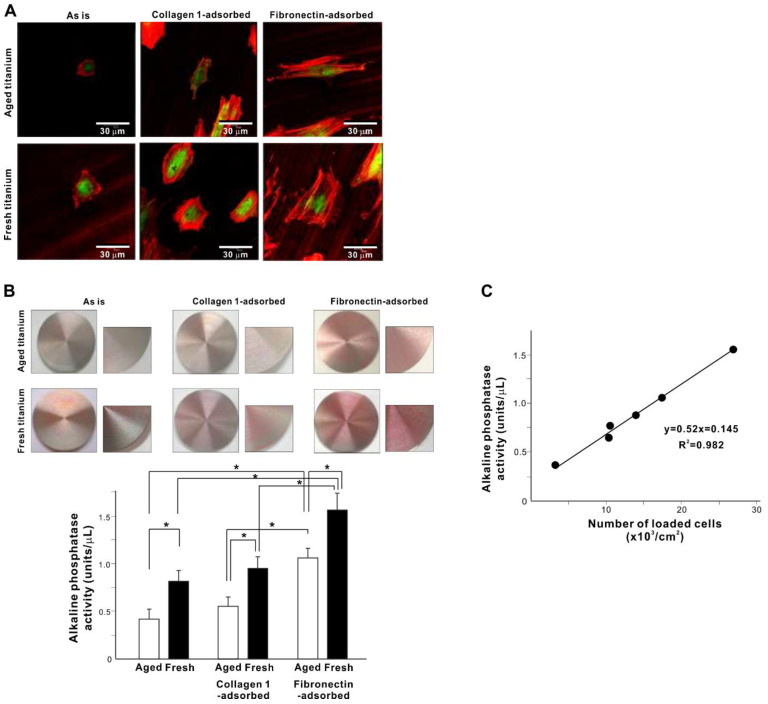
Behavior and function of osteoblasts loaded on various titanium surfaces. (**A**) Confocal fluorescent microscopy images of osteoblasts after a 3-h incubation dual-stained for cytoskeletal actin filaments (red) and focal adhesion protein vinculin (green). (**B**) Alkaline phosphatase (ALP) activity evaluated after three hours of loading onto titanium surfaces. The results of ALP chemical detection are shown in the histogram, with representative ALP-stained images for visual confirmation. * *p* < 0.05, statistically significant difference between two different surfaces. (**C**) Verification of the functional phenotype of loaded osteoblasts. A plot of the number of loaded osteoblasts and ALP activity with the correlation coefficient (R^2^).

Osteoblasts must be healthy and functional after delivery to titanium and contact with mediator proteins if they are to exhibit their desired, bone-forming phenotype. Therefore, we used confocal fluorescence microscopy to examine osteoblast attachment, spreading, and function on the different titanium surfaces 3 h after loading (Figure 5). Although attached osteoblasts were found on all titanium groups, the cells on the aged disks remained small and circular. The cells tended to spread larger on protein-adsorbed disks with stronger expression of cytoskeletal actin and vinculin, a focal adhesion protein (Figure 5A).

After confirming successful osteoblast attachment and cytoskeletal development, we evaluated early-stage osteoblastic function according to alkaline phosphatase (ALP) activity (Figure 5B). ALP activity mirrored the cellular attachment (Figure 4): cells attached to fresh titanium showed higher ALP activity than those attached to aged titanium surfaces regardless of protein pre-adsorption. Fibronectin, but not collagen, significantly increased ALP activity compared with original aged or fresh titanium, both quantitatively and visually. To exclude the possibility of cellular damage or functional degradation during cell loading onto titanium surfaces, the cell-to-function relationship was evaluated by plotting the number of cells loaded and ALP activity (Figure 5C). ALP activity was nearly perfectly correlated with the number of cells, indicating normal manifestation of the functional phenotype and excluding the occurrence of cell damage during loading.

### 3.6. Mechanism of Increased Cell Loading

**Figure 6:**
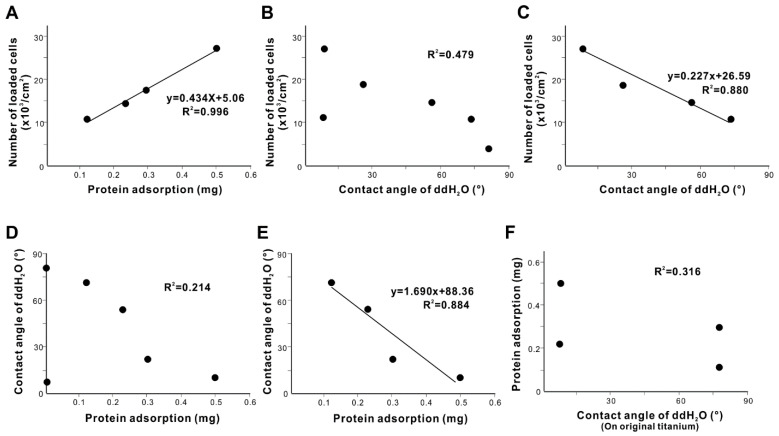
Determinants for cell-loading capability of titanium surfaces. Line graphs show the relationship between the amount of protein adsorption and the number of loaded cells (**A**) and between the contact angle and the number of loaded cells (**B**,**C**). All titanium surfaces were counted in panel (**B**), and only titanium surfaces with protein pre-adsorption were counted in panel (**C**). (**D**,**E**) The determinants for the contact angle. Line graphs show the relationship between the amount of protein adsorption and the ddH_2_O contact angle (**D**,**E**). All titanium surfaces were counted in panel (**D**), and only titanium surfaces with protein pre-adsorption were counted in panel (**E**). Panel (**F**) plots the ddH_2_O contact angle on the original titanium surfaces and the amount of protein adsorption.

We next explored the mechanism underpinning variable cell loading, starting with the associations between protein adsorption efficiency, ddH_2_O contact angle, and number of loaded cells (Figure 6). The number of loaded cells was strongly correlated with protein adsorption (R^2^ = 0.996; Figure 6A). There was no significant correlation between the number of loaded cells and the ddH_2_O contact angle, when all titanium surfaces were counted, regardless of the protein-adsorbed or non-adsorbed titanium (Figure 6B). However, when only titanium surfaces with protein adsorption were tallied, the number of loaded cells was negatively correlated with the contact angle on titanium surfaces with adsorbed proteins (Figure 6C), i.e., more cells loaded onto more hydrophilic surfaces. These results indicate that cell loading depended on both the amount of pre-adsorbed protein and the degree of hydrophilicity of the titanium surfaces. Notably, the R^2^ value was higher for protein adsorption than for the contact angle (Figure 6A,C).

We next investigated the mechanism of hydrophilicity/hydrophobicity. There was no significant correlation between ddH_2_O contact angle and the amount of protein adsorption when titanium surfaces with no protein adsorption were included in the analysis (Figure 6D). However, when only titanium surfaces with protein adsorption were included, the ddH_2_O contact angle was strongly and negatively correlated with the amount of protein adsorption (Figure 6E), i.e., more protein adsorption promoted greater hydrophilicity. Therefore, the ddH_2_O contact angle on titanium was determined by the amount of protein adsorption only on titanium surfaces with protein adsorption. Lastly, we attempted to find a potential determinant of the amount of protein adsorption. There was no significant correlation between protein adsorption and the ddH_2_O contact angle (Figure 6F), disfavoring hydrophilicity/hydrophobicity of titanium as being the primary determinant of protein adsorption efficiency.

## 4. Discussion

**Figure 7:**
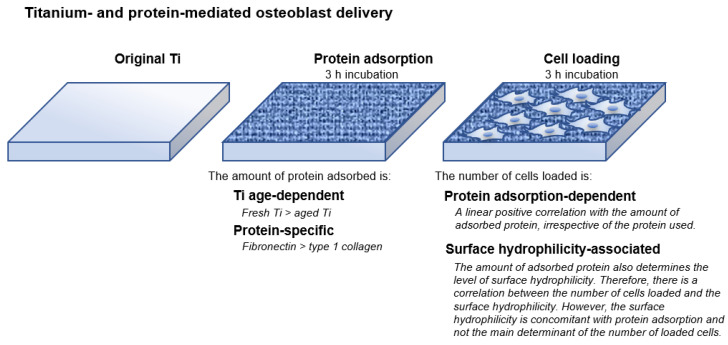
Schematic of the strategy and results of the titanium- and protein-mediated cell delivery system for bone integration, regeneration, and engineering, depicting how to make osteoblast-loaded titanium surfaces effectively and efficiently.

Here we present a simple but effective strategy to make osteoblast-loaded titanium (Figure 7). By understanding how proteins and cells respond to the different properties of titanium, we have developed a novel titanium-mediated osteoblast delivery vehicle applicable to bone integration and regeneration in vivo and ex vivo. To load the maximum number of osteoblasts onto titanium surfaces, we found that proteins must be pre-adsorbed on the surface, with the number of cells successfully loaded onto titanium surfaces directly correlated with the amount of pre-adsorbed protein (Figure 6A). The type of protein did not appear to influence cell loading when considering the two proteins examined here, type 1 collagen and fibronectin. Nevertheless, the efficiency of protein pre-adsorption was specific to the protein, with fibronectin being much more efficient (Figure 2). Both proteins examined in this study are common extracellular matrix proteins harboring Arg-Gly-Asp (RGD) amino-acid sequences that mediate cellular attachment via transmembranous integrins. Other proteins with the RGD peptides, such as vitronectin and laminin, would also be interesting to study.

There are another three significant results from our study. First, protein adsorption was considerably more efficient on fresh titanium surfaces (Figure 2), and this efficiency was not dependent on the hydrophilicity of the fresh surface (Figure 6F). Second, fibronectin adsorbed to titanium surfaces considerably more than type 1 collagen (Figure 2). The role played by surface hydrophobicity/hydrophilicity in material biocompatibility, including for titanium, remains inconclusive, with even the basic hypothesis that more hydrophilic surfaces attach more cells remaining unproven, although some data do seem to suggest this [89,90,96,101,102,127,128,129,130,131]. Although UV-induced superhydrophilic titanium surfaces recruit more cells than non-treated hydrophobic titanium surfaces, there was no linear correlation between the degree of hydrophilicity and the number of cells recruited [127]. The effect of hydrophilicity on protein adsorption is also contentious; some studies have reported that although proteins adsorb strongly to hydrophobic surfaces, proteins adsorbed to hydrophilic surfaces may better attract cells due to better maintained structural and adhesive integrity [132,133,134]. We found that the correlation (R^2^ value) between the contact angle and protein adsorption was only 0.214 (Figure 6D). Other surface properties such as surface carbon and electrostatic charge may also contribute to the biocompatibility of fresh titanium surfaces [96,110,135,136,137]. Fresh titanium surfaces harbor fewer organic impurities and are, therefore, more electropositive than aged surfaces. Indeed, titanium surfaces accumulate carbon-containing molecules from the atmosphere over time, part of the natural biological aging process [95,97,112]. The carbon-containing molecules are primarily hydrocarbons, which are non-polar molecules and electrostatically neutral, masking the innately electropositive titanium surfaces [112]. The effect of surface carbon on protein adsorption to titanium is unknown, but the electropositivity of fresh surfaces may have contributed to the higher protein adsorption because proteins are, in general, negatively charged. It is unclear why fibronectin was more responsive to fresh titanium surfaces. Type 1 collagen and fibronectin are 138 kDa and 400–500 kDa, respectively, and fibronectin seems to react more to the surface properties of biomaterials and modify its form and function by self-assembling into filaments and fibers [133,138]. Regardless, it is crucial that the cell-binding RGD sequence is exposed and functional to recruit and retain cells [134]. Our observation of highly promoted cell loading suggests that the collagen and fibronectin adsorbed to titanium surfaces were not compromised.

The third significant finding was that, even after establishing that surface hydrophilicity was unlikely to determine the degree of protein adsorption (Figure 6F), the protein adsorption and post-protein adsorption contact angle were highly correlated (Figure 6E). As a result, the contact angle and number of loaded cells were correlated (Figure 6C). We hypothesize that protein adsorption is the primary determinant of cell loading and that the contact angle was an additional, associated factor with protein adsorption, because the R^2^ value for protein adsorption was higher than that of the contact angle (Figure 6A,C). Nevertheless, it was significant that surface hydrophilicity was maintained on fresh titanium surfaces or newly acquired on aged titanium surfaces depending on the amount of protein adsorption, which may represent another advantage of these titanium surfaces for subsequent protein-material and cell-material interactions, as the hydrophilicity of fresh titanium surfaces degrades rapidly unless adsorbed with proteins [79,95,97,98,139].

To exclude potential titanium- and mediator protein-driven osteoblast injury, we examined cellular attachment and function immediately after loading. It is critical to deliver healthy and functional cells for bioengineering, and even detaching cells from culture dishes and seeding into new dishes during routine culture can easily compromise cellular viability and function. Incubation for 3 h after seeding is usually not sufficient for cells to spread and settle on biomaterials or even culture-grade polystyrene dishes. Indeed, osteoblasts cultured on aged titanium surfaces with no protein pre-adsorption remained circular after three hours of incubation, without spreading or expressing cytoskeletal actin or the adhesion protein vinculin (Figure 5). However, on fresh titanium and with mediator proteins, the attachment of osteoblasts was accelerated. The osteoblasts spread and expressed actin and vinculin, indicating firm adherence of these cells ready to initiate their function. Notably, these osteoblasts were functional after only three hours of incubation.

ALP activity, a marker of osteoblast function, was highly correlated with the number of loaded cells, irrespective of the adsorbed protein or age of titanium, indicating that the cells were sufficiently functional for cell delivery. Given the promise of this strategy, the UV treatment of titanium, known as the UV photofunctionalization or UV activation, may create similar surface properties to fresh titanium, and applying UV treatment may similarly load titanium with osteoblasts [94,106,140,141,142,143,144,145,146]. Here we tested three hours of incubation for each of protein adsorption and cell loading, i.e., 6 h in total to complete osteoblast-loaded titanium, which could be shortened or optimized in future studies.

The weakness of this study is that the results only apply to bone tissue engineering. Due to its mechanical and non-absorbable nature, titanium may not be suitable for soft tissue engineering. The materials mentioned in the introduction show their advantages for soft tissues. The significance of cell-loaded titanium presented here includes the enhancement of the biomaterial-tissue interface and the avoidance of the need for materials other than titanium. When titanium implants are used as an anchor in bone, bone formation is required at their interface so that the bone and implant are united to work as a load-bearing device, referred to as bone-to-implant integration. The use of fresh titanium and proteins introduced in this study can simply enhance the integration. No material intervention between the titanium and bone would be beneficial to ensure the bone-to-titanium integration. In addition, our approach could easily be applied to other forms of titanium, including but not limited to titanium microfibers and frames, pores, screws, cages, and other implants and scaffolds. Furthermore, other surface topographies than the machined surface should be considered for testing in future studies. The osteoblast phenotypic characterization was limited to ALP activity in the present study. Future studies are expected to conduct more complete characterization of osteoblasts, including the gene expression of bone-related proteins and growth factors and the calcium deposition. It should also be noted that the proliferative activity and adhesion strength of cells were not directly evaluated in the present study. Although the ALP activity may represent the total phenotypic outcome reflected by the level of differentiation and proliferation of osteoblasts, a direct measurement of cell proliferation should be considered. Although cell spreading behavior was qualitatively evaluated by the expression of cytoskeletal actin and adhesion protein, future studies need to determine if the use of fresh titanium and the mediator proteins increases the adhesion strength of cells on titanium so that the cells are more resistant to exogenous force. In vivo studies are then encouraged to further develop the technology. Cell-loaded cylindrical titanium implants would be suitable for the first in vivo animal study, followed by testing titanium microfiber scaffolds on which cell attachment is considered very challenging. We anticipate the synergy effect of fresh titanium and fibronectin will promote cell-loading on titanium and thereby accelerate and enhance bone integration and generation.

## 5. Conclusions

Here we developed an effective and efficient approach to make osteoblast-loaded titanium for bone integration and regeneration. Loading osteoblasts was most efficient on fresh rather than aged titanium adsorbed with fibronectin as a cell attachment mediator. The number of osteoblasts loaded using this synergistic approach was nine times greater than that on baseline, aged titanium with no protein adsorption. Osteoblast loading was completed in six hours (three hours for protein adsorption and three hours for cell loading), and the loaded cells were confirmed to be attached and functional. Furthermore, the number of loaded cells was strongly correlated with the degree of mediator-protein adsorption, regardless of the type of protein, with fibronectin more efficiently adsorbed to titanium surfaces than collagen 1. It remains unclear whether the surface hydrophilicity exclusively found on fresh titanium surfaces caused the increased protein adsorption or cell loading. Interestingly, the hydrophilicity of protein-adsorbed titanium increased with the amount of protein adsorption, but the induced hydrophilicity alone did not determine cell loading efficiency.

## Data Availability

Data availability on request from the authors.
